# Supplementary material for: Hydrolysis-dependent severing tunes internal monomeric heterogeneity to shape actin length distributions
Source: bioRxiv. 2025 May 30:2025.05.29.656816. Preprint. [Version 1] doi: 10.1101/2025.05.29.656816 (PMC12154627; doi:10.1101/2025.05.29.656816)
Supplement: 1 [file NIHPP2025.05.29.656816V1-supplement-1.pdf]

bioRxiv preprint doi: https://doi.org/10.1101/2025.05.29.656816; this version posted May 30, 2025. The copyright holder for this preprint (which was not certified by peer review) is the author/funder, who has granted bioRxiv a license to display the preprint in perpetuity. It is made available under aCC-BY 4.0 International license.

Supplementary Information: Hydrolysis-dependent severing tunes internal monomeric heterogeneity to shape actin length distributions

Soumyadipta Ray[1*#], Binayak Banerjee[2#], Lishibanya Mohapatra[1*], and Dipjyoti Das[2*]

[1]School of Physics and Astronomy, College of Science, Rochester Institute of Technology, Rochester, NY 14623, USA
[2]Department of Biological Sciences, Indian Institute of Science Education And Research Kolkata, Mohanpur, Nadia - 741 246 West Bengal, India.
[*]Correspondence: sorsps@rit.edu, lxmsps@rit.edu, dipjyoti.das@iiserkol.ac.in
[#] contributed equally

S1 CALCULATION OF P_n

Here we have used a mean field mathematical framework following previous studies (1–4). We first define a variable P_n as the probability that the n-th monomer is undecorated, and hence $(1 - P_n)$ is the probability that the n-th monomer is decorated. We can then write down the time evolution equations (Master equations) of these state probabilities and solve them to obtain an analytical expression for P_n. Thus, the Master equations for P_n would be,

for $n = 1$,

$$\frac{dP_1}{dt} = k_{growth}(1 - P_1) - k_{eff}P_1 \tag{S1}$$

and, for $n \geq 2$,

$$\frac{dP_n}{dt} = k_{growth}\left[P_{n-1}(1 - P_n) - (1 - P_{n-1})P_n\right] - k_{eff}P_n. \tag{S2}$$

These equations are written by collecting the ‘gain’ and ‘loss’ terms for the probabilities. Also, note that adding an ADP-Pi monomer via polymerization shifts the site indices in the terms since the reference frame for counting the n-th site starts from the barbed end. For instance, in Eq. (S2), the addition of an ADP-pi monomer at the barbed end can positively contribute to the n-th site if the configuration was $P_{n-1}(1 - P_n)$. However, it negatively contributes to P_n if it was $(1 - P_{n-1})P_n$ before adding a monomer. Note that, k_{eff} is the coarse-grained effective switching rate from an undecorated (ADP-pi or ADP) to a decorated (ADP-actin-cof) monomer. Additionally, this switching always changes the n-th site from P_n to $(1 - P_n)$.

In the steady state, the above equations (Eqs. S1 and S2) reduce to $P_1 = \frac{k_{growth}}{k_{growth}+k_{eff}}$ and $P_n = \frac{k_{growth}}{k_{growth}+k_{eff}}P_{n-1}$. Therefore, we obtain

$$P_n = \left(\frac{k_{growth}}{k_{growth} + k_{eff}}\right)^n. \tag{S3}$$

Note that P_n is a geometric distribution.

bioRxiv preprint doi: https://doi.org/10.1101/2025.05.29.656816; this version posted May 30, 2025. The copyright holder for this preprint (which was not certified by peer review) is the author/funder, who has granted bioRxiv a license to display the preprint in perpetuity. It is made available under aCC-BY 4.0 International license.

S2 SUPPLEMENTAL FIGURES

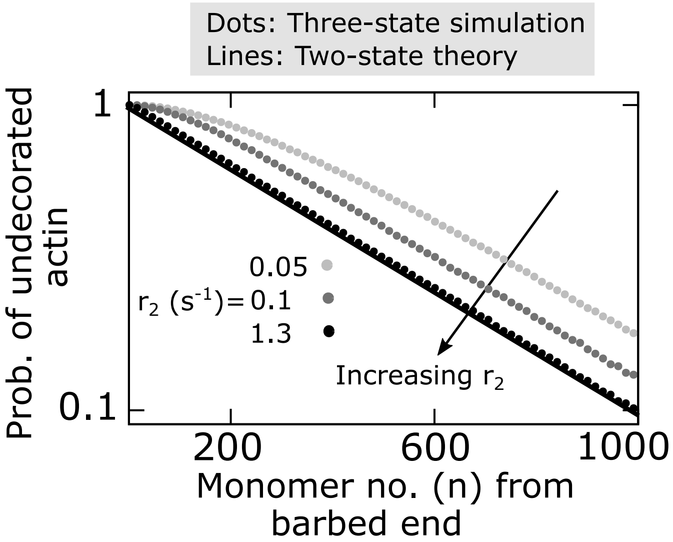

Figure S1: The probability of undecorated-actin within a filament as a function of monomer number from the barbed end, shown for different values of cofilin-dependent Pi release rate r_2. Analytical curves from the two-state model are shown in lines, simulation from the three-state are represented by dots. Parameters: $[actin] = 0.8\mu M$, $[cof] = 0.6\mu M$, $k_{b0} = 0.15s^{-1}$, $R = 100$. Other parameters: Table 1.

REFERENCES

1. Stukalin, E. B., and A. B. Kolomeisky, 2006. ATP hydrolysis stimulates large length fluctuations in single actin filaments. Biophysical journal 90:2673–2685.

2. Keiser, T., A. Schiller, and A. Wegner, 1986. Nonlinear increase of elongation rate of actin filaments with actin monomer concentration. Biochemistry 25:4899–4906.

3. Niedermayer, T., and R. Lipowsky, 2015. Association-dissociation process with aging subunits: Recursive solution. Physical Review E 92:052137.

4. Ranjith, P., K. Mallick, J.-F. Joanny, and D. Lacoste, 2010. Role of ATP-hydrolysis in the dynamics of a single actin filament. Biophysical journal 98:1418–1427.
